# Politics of a Director of Radiology

**DOI:** 10.2349/biij.5.4.e23

**Published:** 2009-10-01

**Authors:** M Soo

**Affiliations:** Westmead Hospital, New South Wales, Australia

In my formative years as a radiologist working at the Lysholm Department of Neuroradiology in London, I committed the cardinal sin of injecting air into the subdural space when performing a pneumoencephalogram. The consultant supervising the procedure considered the examination a failure. I was embarrassed yet annoyed because the quality of the radiographs was quite satisfactory. At a reporting session the following day, Dr. James Bull, the world-renowned Head of our Department, concluded that my ‘failed’ pneumoencephalogram was normal. This incident in 1967 gave me a glimpse of my chief’s exemplary character — someone who stood by his trainee in his hour of need. Dr. Bull’s immense intellect and strong personality brought his department worldwide fame; his staff were loyal and happy, a clear case of a Director operating in an ideal world.

Technological advances in recent decades have revolutionised the practice of radiology. The computer manager and clerical coordinator have moved into the core of departmental management. Time and personnel resources are demanded of radiology because of the growing numbers of clinical interface. Society’s move towards egalitarianism means it is acceptable to put a young and industrious person in charge. Such a Director might be a novice but may have been appointed by the higher echelon of administration for political reasons. Of the many unseen agenda items waiting at the Director’s office, none are as crucial as the politics of running a department. Indeed, ‘politicking’ is a prime if unspecified job specification of the Director. There is no guaranteed crash course in people management in radiology. Much like a round of golf, politics in radiology is a learn-as-you-go thinking process. The talented are quicker to succeed; the journeyman takes a little longer.

Regardless of age or experience, the Director of radiology is the public face of the department. Patients, the media and clinical colleagues perceive the department through the actions or inactions of its Head. The various facets of the Director’s personality are reflected by his or her compassion, coolness under fire and willingness to go first on-call for interventional procedures — a leader by example. Yet in the odd instance, a Director might enjoy patrolling the reporting room, making sure all radiographs are reported. Small wonder then that this person is remembered not only as a competent radiologist, but also as a policeman.

Loyalty is a cherished quality that works both ways. An astute Director shows loyalty, respect and tolerance towards staff members regardless of their calling. The divisive influence of disloyalty must be prevented at all costs lest it leads to gossip, bad-mouthing, and unfounded accusations. These are the ingredients that cause unnecessary conflicts that the Director must resolve. Should the problem be handed over to the Human Relations Unit? Heads of department will do well to make understanding human behaviour their hobby. I treat an altercation the way I write up an interesting case study — to be filed for future reference.

Well-meaning vendors are keen to sell state-of-the-art machines if only to raise their company’s profile. It bodes well for the Director to have a working knowledge of the specifications and installation costs of modern radiological equipment. An offer of a study grant to staff is occasionally made. Beware of Greeks bearing gifts! Show no hint of conflict of interests in such dealings. The line between right and wrong can be ill-defined. The grace to accept or reject such offers and still retain the company’s goodwill falls within the prerogative of a shrewd Director.

One of the sternest tests for a Director in hospital practice is dealing with vascular surgeons who want to perform procedures on their patients in the department’s angiographic suites. Their bargaining point is not to refer cases if their wishes are not met. Disappointingly, radiology lacks the manpower to compete as the majority of newly qualified imaging specialists prefer to be diagnosticians rather than interventionists. What choice is there for the Director should hospital administration be on the side of the surgeons? The wisdom of peaceful coexistence applies in this context: an offer of a session or two seems a reasonable compromise. Turf battles are the bane of radiology in modern times. It behoves the Director to be sensitive to the ambitions of some clinicians.

By virtual of their numbers, radiographers are a pressure group. To a lesser extent this holds true with the nurses. In the Australian community, nurses regard themselves as patients’ advocates. Such an attitude can be viewed as constructive. Pressure groups see the work ethics of the radiologists from a different perspective. And it is their collective opinion of the department’s activities that a street-wise Director should take seriously.

The dwindling health-care budget has spawned an increasing number of professionally trained administrators. Meet this sage whom we call the business manager. Their briefs are to oversee and advice on how to run a busy department on a stringent budget. The business manager is the Director’s friend, partner and occasional saviour. Is he or she the guru or a usurper of power? Consider this: it is invariably the business manager’s job to advise and assist the Head when it comes to writing a ‘business case’ for the purchase of equipment.

When it comes to assessing the Director’s performance, it is the responsibility of a committee that consists of the CEO and most certainly a senior clinical academic. This high profile senior clinician could have once voted in favour of the Director. The sad truth is that radiology as a discipline has not entirely escaped from the cultural law of being influenced by doctors from the clinical streams. But a strong Director will pursue his or her own agenda. Therefore, despite the back-stabbing and cynicism from some quarters, to be appointed Director is to reach the pinnacle of the profession. Every radiologist should aspire to be Head for at least a four-year term. It not only gives one an insight into the job’s complexities but also the quirkiness of human nature. You will be a better person for having done so.

